# Resin-Reinforced Auxetic Structures with Re-Entrant Struts for Improved Energy Absorption

**DOI:** 10.3390/polym17233176

**Published:** 2025-11-29

**Authors:** César Garrido, Jorge Fernández, Víctor Tuninetti, Gonzalo Pincheira, Ignacio Ríos, Rodrigo Valle

**Affiliations:** 1 Department of Mechanical Engineering, Universidad del Bío-Bío, Concepción 4081112, Chile; cgarrido@ubiobio.cl (C.G.); jfernandez@ubiobio.cl (J.F.); 2 Department of Mechanical Engineering, Universidad de La Frontera, Temuco 4811230, Chile; victor.tuninetti@ufrontera.cl; 3 Department of Industrial Technologies, Faculty of Engineering, University of Talca, Camino a Los Niches Km 1, Curicó 3344158, Chile; gpincheira@utalca.cl; 4 Master Program in Engineering Sciences, Faculty of Engineering, Universidad de La Frontera, Temuco 4811230, Chile; 5 Construction Multidisciplinary Research Group, Facultad de Arquitectura, Construcción y Medio Ambiente, Universidad Autónoma de Chile, Talca 3460000, Chile

**Keywords:** mechanical characterization, auxetic atructures, cellular structures, energy absorption

## Abstract

This study aims to improve the energy absorption and mechanical properties of auxetic structures by optimizing their design and analyzing the influence of different resin matrices in composite fabrication. Auxetic materials exhibit unique deformation behavior due to their negative Poisson’s ratio, making them promising for energy absorption applications. However, their practical implementation is often constrained by their intrinsic mechanical properties, particularly their strength under realistic loading conditions. This research investigates the effect of resin matrix selection on the energy absorption capacity, Young’s modulus, and yield strength of auxetic composites. A systematic experimental campaign was conducted, subjecting auxetic structures reinforced with various resin matrices to compressive loading. The results indicate that embedding the auxetic structure within a resin matrix significantly enhances energy absorption compared to pure resin samples. Notably, vinylester resin composites exhibited the highest Young’s modulus, yield strength, and energy absorption capacity. This superior performance is attributed to the synergistic interaction between the auxetic structure, which efficiently distributes stress, as well as the intrinsic toughness and load-bearing capacity of the vinylester resin. These findings contribute to the optimization of auxetic composites for energy absorption applications and the development of high-performance materials for impact mitigation in aerospace and automotive industries.

## 1. Introduction

Composite metamaterials in mechanics arise from the integration of microstructures with various materials to meet specific property demands. Technological advances in industry have spurred continuous innovations in materials and manufacturing methods [1]. Significant progress has facilitated the development of additive manufacturing (AM), allowing the production of components with intricate geometries on different scales and materials [2], allowing 3D printing processes to be incorporated into building construction balancing thermal and structural properties [3]. In the same way, a notable outcome of these advances is the creation of microstructured materials, often referred to as cellular structures [4]. These structures are composed of struts and nodes interconnected in a periodic pattern [5], and their geometry can be customized to control the mechanical behavior of the macrostructure [6,7]. The topology of these structures is typically designed and optimized based on the desired mechanical properties [8,9], sometimes drawing inspiration from natural sources; for example, ref. [10] explored structures that mimic the configuration of trabecular bone. His research demonstrated that these structures offered superior energy absorption compared to traditional designs. Similarly, in [11], a 3D-printed curved honeycomb is proposed with analytical formulations to predict mechanical properties. The design provides optimal configurations in stiffness and plateau stress, which could have significant applications in lightweight materials engineering. In [12], biomimetic network design strategies inspired by natural structures capable of combining high rigidity with efficient energy dissipation were reviewed. Along with growing research, a solid foundation has been laid for the engineering of high-performance auxetic composites through numerical modeling of 3D-printed lattices, allowing for better prediction of their deformation mechanisms [13].

The geometric flexibility provided by AM enables the creation of cellular structures with mechanical properties superior to those of traditional materials [14], such as high strength and stiffness combined with low weight, and even structures exhibiting a negative Poisson’s ratio [15,16]. This design strategy is even used to print aerated concrete, where [17,18] demonstrated that the structures of interconnected gyroid cells and gaps showed a significant increase in compressive strength. The design of cell structures for specific mechanical properties can be carried out using analytical methods [19,20,21]. Thus, these design approaches have allowed the development of materials that contract laterally upon stretching, auxetic structures that expand laterally under tension, or contract laterally under uniaxial compression, exhibiting a negative Poisson’s ratio [22]. This auxetic effect results in desirable properties such as excellent indentation resistance [23,24], high shear stiffness [24,25], remarkable fracture toughness [22], and unique acoustic energy absorption capabilities [26,27]. Recent experimental studies have validated this mechanism in polymer networks manufactured using additive manufacturing, confirming the typical behavior of the negative Poisson’s ratio [28]. Research on auxetic structures has shown promising results in deformation enhancement and energy absorption. In [29], a three-dimensional structure with zero Poisson ratios was developed, confirming its effectiveness through compression tests and numerical simulations, suggesting applications in advanced materials design. The authors of [30] investigated the compression response and energy absorption of 3D-printed Kelvin polymer foams. Four deformation modes were identified and a relationship between relative density and energy absorption behavior was established, highlighting the importance of loading rate. In [31], a novel hierarchical triangular honeycomb designed for excellent energy absorption is introduced, highlighting its superior performance compared to conventional hexagonal honeycombs. Ref. [32] focuses on 3D-printed sandwich panels with a core inspired by biological structures, revealing that asymmetric configurations offer better energy dissipation. Among auxetic structures, those with re-entrant struts are notable for their extraordinary energy absorption capabilities under quasistatic and low-velocity impact loads [22,33,34]. Initial studies by Lakes (1987) [35] and Evans (1989) [36] laid the groundwork by proposing geometric configurations with re-entrant struts and developing analytical approaches to predict transverse expansion under longitudinal load. Recent research has employed various theoretical design approaches to analyze the mechanical behavior of re-entrant struts, using geometric parameters to manipulate Poisson’s ratio and Young’s modulus. Timoshenko’s classical theory has been effective in predicting the elastic properties of these structures [37,38,39]. For example, ref. [40] designs a new re-entrant structure by adding wedge-shaped pieces, enhancing structural stiffness during compression and preventing lateral buckling, thus ensuring more stable auxetic behavior. Furthermore, ref. [41] proposes an auxetic honeycomb for a sandwich structure with a novel stepped design, achieving a graduated auxetic design by varying the angle of the honeycomb cell through the core thickness. This results in increased bending failure stress and specific energy absorption. Studies like [42,43] further explore new re-entrant auxetic structures, demonstrating improvements in specific modulus, specific resistance, and specific energy absorption.

Academic interest in auxetic metamaterials has grown exponentially over the past five years, reflecting a shift toward multidisciplinary research focused on practical applications [44]. In this way, auxetic structures exhibit great potential for engineering applications. However, due to the low mechanical resistance resulting from weak interlayer adhesion inherent to fused deposition modeling (FDM) technologies, auxetic structures fabricated through this method demonstrate limited energy absorption capacity, which significantly restricts their use in real-world engineering scenarios. To overcome this limitation, several studies have proposed reinforcement strategies that transform these geometries into composite materials with enhanced properties. One of the most extensively explored approaches is the use of polymeric foams as filler materials. For example, Airoldi et al. [45] demonstrated that the incorporation of soft open-cell polyurethane foam into 3D-printed hexa-chiral auxetic frames significantly improves energy absorption, generating a synergistic effect greater than the sum of the individual components. Széles et al. (2024) and Zhang et al. (2024) demonstrated that these configurations outperform conventional honeycomb architectures in impact and compression tests, achieving higher plateau stresses and specific energy absorption [46,47]. Baroutaji et al. (2024) demonstrated that graduated recessed honeycombs can be optimized to achieve specific stiffness and energy absorption characteristics by adjusting the cell angle, gradient, or wall thickness [48]. Széles et al. (2024b) proposed double re-entrant topologies optimized to suppress buckling instabilities and improve deformation stability under compression [46]. Similarly, in scenarios involving combined shock and impact loads, such as ballistic applications, Zhao et al. [49] analyzed flexible composite structures that combine graded and auxetic foams, concluding that these configurations are highly effective in mitigating compound loads (shockwaves and fragments), outperforming conventional materials in terms of energy dissipation. NamdariPour et al. [50] investigated re-entrant auxetic structures filled with polymethyl methacrylate (PMMA) foam reinforced with carbon nanotubes (CNTs), reporting a 75% increase in peak fracture strength and up to a 130% improvement in energy absorption, along with enhanced post-peak stability. Likewise, Liu et al. [51] examined the ballistic performance of foam-filled auxetic structures and recorded a 6.12% increase in ballistic limit velocity and stabilization of specific energy absorption (SEA) values around 550 [J/kg]. In a comprehensive review, Gomes et al. [52] analyzed over one hundred studies focused on tubular auxetic structures and highlighted the high efficiency of polyurethane foams in various engineering applications, ranging from biomedical devices to aerospace energy absorbers. Concurrently, Zhao et al. [53] explored the integration of aluminum foams into carbon-fiber-reinforced polymer (CFRP) auxetic structures, observing significant enhancements in elastic modulus, plateau stress, and SEA, which supports their application in lightweight energy-absorbing components for automotive and aerospace sectors. From an applications perspective, Bozorgnia-Tabary et al. (2024) demonstrated the feasibility of using auxetic composites based on recycled PLA for impact protection, highlighting their environmental and mechanical advantages [54]. In this way, additive manufacturing (AM) enables the precise fabrication of complex auxetic lattices across multiple scales and material systems, expanding their design possibilities beyond traditional geometries [55].

Another relevant line of research involves the use of cementitious matrices as reinforcement media. Meng et al. [56] embedded 3D-printed auxetic networks into cement-based mortars and showed that these geometries redistributed internal stresses more effectively and reduced crack propagation, resulting in a 1.7 fold increase in densification energy compared to conventional composites. Wu et al. [57] applied 3D-printed auxetic structures to improve the fracture resistance of cemented backfill in underground mining operations, achieving up to 30.66% improvements in post-peak load capacity and toughness. Although Genç et al. (2024) and Hematibahar et al. (2024) have demonstrated the feasibility of auxetic reinforcements in cementitious matrices, studies on polymeric or resin-based auxetic composites remain limited [58,59]. Castro et al. (2025) further explored the application of reentrant networks under cyclic loads, highlighting their recovery and damping capabilities, suggesting their potential use in seismic protection systems [60]. Meanwhile, Momoh et al. [61] provided a state-of-the-art review on the integration of auxetic geometries into cementitious matrices, noting that although promising benefits have been demonstrated in laboratory-scale specimens, very few studies have advanced toward full-scale structural applications. Similarly, in the field of polymeric resin reinforcement, Li et al. [62] infiltrated additively manufactured alumina microstructures with epoxy resin, achieving high specific strength values (113.5–142.6 [MPa]) and specific energy absorption (25.3–35.6 [J/gr]) at relatively low densities. Xue et al. [63] reinforced tubular auxetic structures with epoxy, reporting delayed crack initiation, more stable collapse mechanisms, and improved structural durability. In another study, Xue et al. [64] used pressure-assisted infiltration of photosensitive resin into aluminum auxetic structures, obtaining notable improvements in stiffness, compressive strength, and energy absorption due to efficient interaction between the struts and the filler material. Likewise, Li et al. [65] demonstrated that elastomeric reinforcement using glassy polymers in auxetic designs significantly enhances stiffness and energy absorption, and that the magnitude of improvement increases with more negative Poisson’s ratios. Furthermore, research has shown that reinforcing an asymmetric auxetic structure with polyester resin, taking advantage of the manipulable factor of asymmetry, significantly improves the energy absorption capacity. However, the impact of the different types of resin on the mechanical functionality of these auxetic structures remains poorly studied.

Reinforcing auxetic lattices with foams, cementitious materials, or polymeric resins effectively preserves their deformation mechanisms while significantly improving stiffness, toughness, and energy absorption. Among these strategies, the use of rigid polymer resins is particularly attractive because the resin can be directly infiltrated into the lattice without the need for complex tooling or molding procedures. However, despite increasing interest in resin-filled auxetic composites, prior research mainly focuses on a single resin system, typically epoxy, and often reports improvements based on only one mechanical indicator. A comprehensive comparative assessment that quantifies the influence of different industrial resins on Young’s modulus, yield strength, and energy absorption capacity has not yet been performed. To address this gap, the present work experimentally evaluates auxetic structures reinforced with epoxy, polyester, and vinylester matrices and determines how resin selection governs the load bearing and energy absorbing response, with the goal of supporting informed material design for lightweight protective and structural applications.

The subsequent sections of this paper are organized as follows. Section 2 describes the materials used, auxetic structure fabrication, resin reinforcement procedures, and quasi-static compression testing methodology. Section 3 presents and discusses the experimental results, including the influence of resin type on stiffness, yield behavior, and energy absorption performance. Finally, Section 4 summarizes the key findings of this study and identifies opportunities for future research on resin-reinforced auxetic composites.

## 2. Materials and Methods

### 2.1. Auxetic Structures Manufactured with Additives

**Figure 1:**
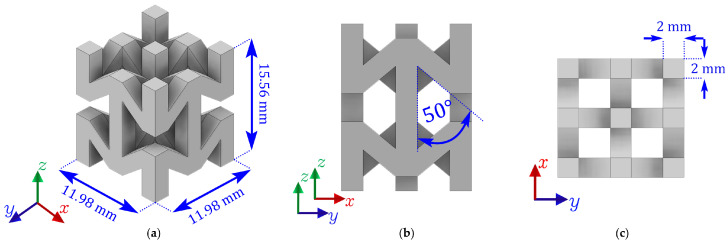
(**a**) Tridimensional unit cell of the re-entrant auxetic structure, (**b**) Side view of the unit cell illustrating the re-entrant angle θ=50° and (**c**) Top view of the unit cell layout, highlighting the periodic arrangement and 2 mm thickness of the struts.

In this study, a classic auxetic structure with re-entrant struts has been used to evaluate the energy absorption capacity combined with different polymer matrices. According to the latest studies [66,67,68] structures with a re-entrant angle θ=50° experience greater ductility. Similarly, in [69], the great energy absorption capacity that this type of re-entrant auxetic cell can achieve has been demonstrated. Therefore, in this study the base unit cell has been designed with a re-entrant angle of θ=50° to achieve higher levels of energy absorption. The dimensions of the unit cell are shown in Figure 1.

**Figure 2:**
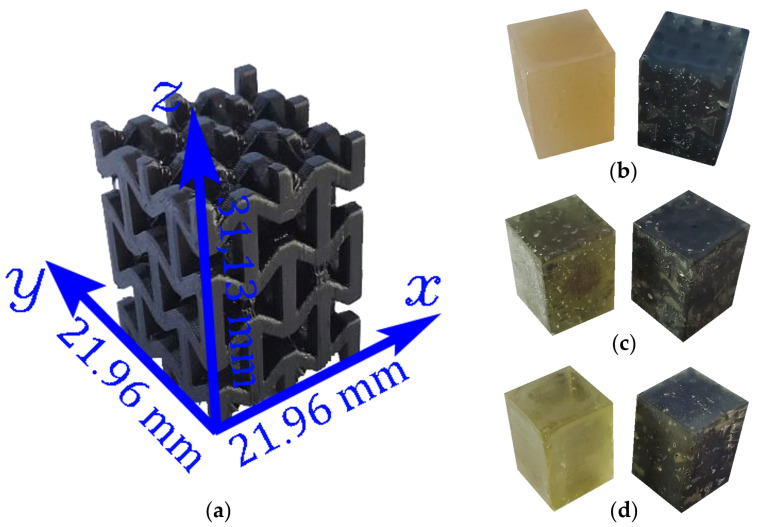
Auxetic lattice manufactured by FDM using PLA, showing coordinate axes and external dimensions (**a**). Polyester-based samples (**b**), Vinylester-based samples (**c**), and Epoxy-based samples (**d**), each including a neat resin cube and a corresponding resin-filled auxetic composite for quasi-static compression testing.

Through this unit cell, three types of experimental samples were manufactured. On the one hand, macrostructures of 2×2×2 unit cells were fabricated for quasi-static compression experiments, and their dimensions are shown in Figure 2. The structures were manufactured with an Ender 3 fused filament fabrication (FFF) system with a 1.75 mm diameter PLA filament from the ANET brand. The printing was carried out on the x,z plane to avoid the need for support structures. Material extrusion was performed at a constant temperature of 220 °C through a 0.4 mm nozzle, while the print bed temperature was maintained at 50 °C and the layer height was set to 0.2 mm. A 100 % linear infill pattern was used. On the other hand, to improve the load-bearing capacity of the structures and to evaluate their energy absorption performance, each structure was filled with one of three types of resin: [Resin 1—Polyester Resin, FibraTec, Santiago-Chile], [Resin 2—Vinylester Resin, FibraTec, Santiago-Chile], and [Resin 3—Epoxy Resin, PlastiQuimica, Santiago-Chile]. Likewise, for each case a sample of pure resin was manufactured to compare the mechanical behavior with the composite structure, as shown in Figure 2. For each case, two experimental samples were manufactured, and the resulting mechanical properties presented in this work represent the mean values obtained from these two samples. The complete count of manufactured samples for all configurations is summarized in Table 1. The use of two specimens per configuration was selected due to the high fabrication effort and exploratory nature of the study; however, the consistent trends observed support the reliability of the results.

**Figure 3:**
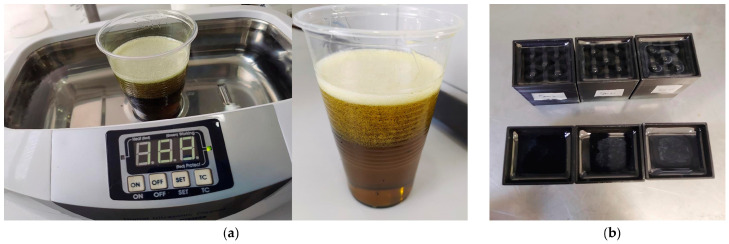
Cleaning process in an ultrasound system to eliminate bubbles in the resin (**a**); the samples are then poured into the molds (**b**).

The composite structure manufacturing process was carried out using 3D printing-made molds with the same dimensions of the structures, where the resin was later poured. On the other hand, Polyester and Vinylester resins contain 2% of cobalt, which acts as a drying accelerator in the resin, helping to achieve good surface drying. In both cases, the proportion between the resin and the hardener is 2 cc per 100 g of resin. In addition, the epoxy resin does not have a percentage of cobalt added, and the mixing ratio between resin and catalyst is 2:1. That is, for a 100 g mixture, 66.6 g of resin and 33.3 g of hardener must be added. Once the resin was mixed with its respective catalyst, the mixture was processed in an ultrasonic cleaner for 5 min to eliminate bubbles, as shown in Figure 3.

It is important to mention that all experimental samples were manufactured on the same day and in the same batch. Subsequently, the resin samples were demolded after 24 h, and the experiments were carried out on the same day after 7 days. Therefore, all experimental resin samples have the same curing time.

### 2.2. Experimental Testing

Once the experimental samples were built, quasi-static compression experiments were carried out to evaluate the energy absorption behavior of each configuration. A Zwick Roell Z100 testing machine (Santiago, Chile) with a 100 kN capacity was used, operating within a crosshead speed range of 0.0005 mm/min to 300 mm/min. For the compression experiments, ASTM D-695 was followed for specimen alignment, loading configuration, and strain-rate control. Accordingly, a constant deformation speed of 1 mm/min was applied throughout the test.

**Figure 4:**
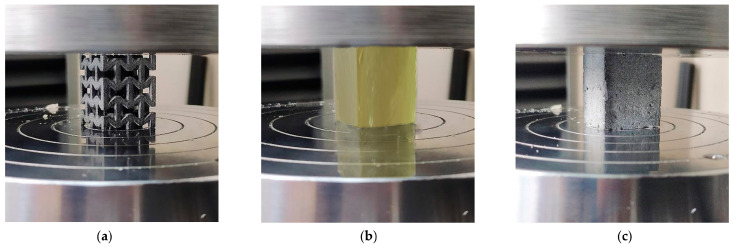
Quasi-static compression experiments in: Auxetic structure (**a**), Resin (**b**) and Composite structure (**c**).

To acquire the experimental results, the test machine continuously recorded load versus crosshead displacement data. Figure 4 presents the experimental setup for the quasi-static compression tests, including the auxetic structure, the cured resin sample and the resulting composite configuration.

Although ASTM D-695 is typically applied to determine compressive properties within the elastic and proportional regions, the tests in this study were extended to characterize the large-strain energy absorption response of the auxetic structures. Therefore, loading was continued beyond the proportional limit until the first occurrence of visible failure initiation or a maximum plastic strain of approximately 60%, whichever occurred first.

### 2.3. Specific Energy Absorption

The literature suggests that auxetic structures with a negative Poisson’s ratio have the potential to enhance their energy absorption capacity under plastic deformation [21,70,71]. Using an auxetic structure, one establishes conditions for energy absorption that extend from the final stage of elastic behavior to the onset of densification. This interval, known as the energy absorption plateau region, appears in the force or stress response subsequent to the yielding point of the structure designed for energy absorption. During this phase, the force or stress remains relatively constant or nearly constant, while deformation gradually increases. The energy absorbing structure thus dissipates maximum energy without a significant rise in force or stress. Consequently, the plateau region plays a critical role in the response curve of such structures, ensuring effective and safe energy dissipation. The expression for estimating engineering stress is derived from the energy per unit volume, as illustrated in Equation (1).

(1) $$\sigma_{p}=\frac{W_{p}}{\Delta_{e}}=\int_{\varepsilon_{y}}^{\varepsilon_{d}}\sigma \left(\varepsilon \right)d\varepsilon$$

The parameter $\sigma_{p}$ represents the area under the stress curve between the points $\varepsilon_{y}$ and $\varepsilon_{d}$. To determine the initial point $\varepsilon_{y}$ of the energy absorption zone, the criterion used is the conclusion of the elastic proportional behavior of the structure or resin-filled structure. The end point is established when a significant progression in the horizontal response of the structure, referred to as the energy absorption zone, transitions to a noticeable vertical trend indicating an abrupt increase in stress. To compare the energy absorption capacity of auxetic structures with resin reinforced structures, the Specific Energy Absorption (SEA) will be calculated as cited in [21], as shown in Equation (2).

(2) $$SEA=\frac{1}{\rho }\times \int_{\varepsilon_{y}}^{\varepsilon_{d}}\sigma \left(\varepsilon \right)d\varepsilon$$

where SEA is the specific energy absorption, ρ the specific density of the characterized element, and σ the plateau stress between two engineering strain points $\varepsilon_{y}$ and $\varepsilon_{d}$. To ensure consistency in the SEA estimation, the yield strain $\varepsilon_{y}$ was defined using the conventional 0.2% offset method applied to the linear elastic region of each stress–strain curve. The densification strain $\varepsilon_{d}$ was defined as the first strain at which the engineering stress increased by at least 15% relative to the plateau stress level, indicating the onset of structural densification. These quantitative criteria were applied uniformly to all samples to ensure reproducibility and comparability of SEA values across different material systems. The following section presents the experimental results for auxetic structures fabricated with PLA and structures reinforced with the three different types of resin, analyzing the SEA capacity for each case.

## 3. Results and Discussion

**Figure 5:**
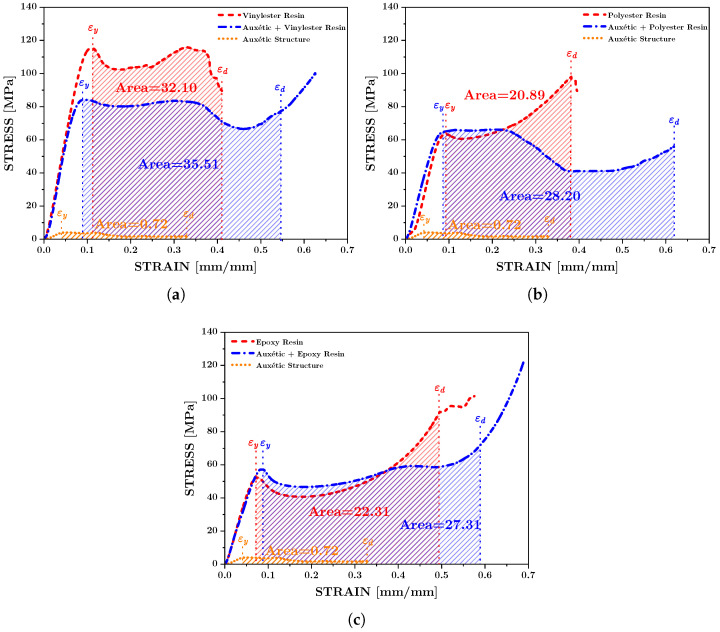
Experimental engineering stress and strains for each auxetic structure reinforced with vinylester resin (**a**), polyester resin (**b**) and epoxy resin (**c**).

The quasi-static compression response for all tested configurations is presented in Figure 5, including the auxetic structure reinforced with Vinylester, Polyester, and Epoxy resins. The curves demonstrate consistent behavior between specimens within each material system, confirming that the averaged results are representative of each configuration.

The deformation capacity of each configuration can also be compared based on the experimental stress–strain response in Figure 5. The auxetic structure exhibited lower strength but sustained large strains before failure due to its cellular architecture. In contrast, the neat resins showed higher stiffness and strength but failed at relatively lower strains. When the auxetic structure was embedded within the resin matrix, the resulting composite structures combined the advantages of both constituents, exhibiting improved strength while still maintaining significant plastic deformation. This demonstrates that the auxetic reinforcement enables a more gradual and stable collapse mechanism, contributing to increased energy dissipation under compressive loading.

It is noted that two specimens were tested per condition; however, the stress–strain curves exhibited minimal scatter and the calculated mechanical properties showed a coefficient of variation below 8%, indicating that the performance differences among the matrix systems substantially exceed the experimental variability and support the robustness of the reported trends.

**Figure 6:**
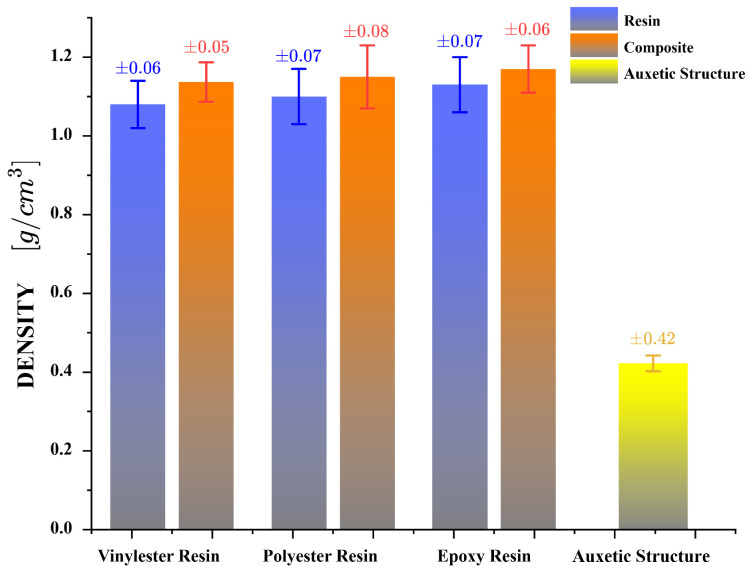
Measured density for each experimental sample under quasi-static compression.

Figure 6 shows the measured density of each experimental sample, evidencing that the inclusion of the auxetic structure slightly increases the density compared to the neat resins, due to the addition of solid structural reinforcement.

**Figure 7:**
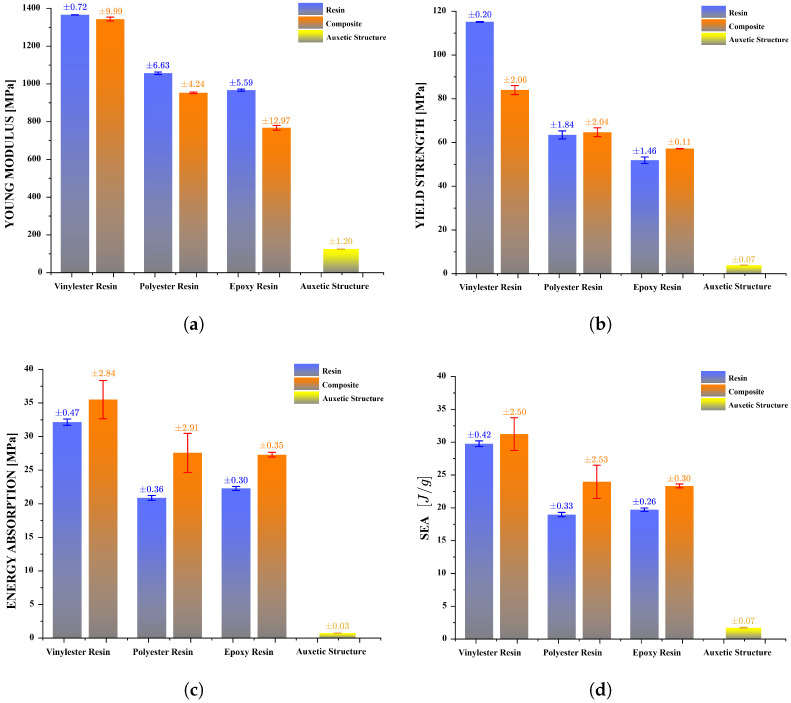
Analysis of experimental results under compression, in which the mechanical behavior of each type of resin is compared with the composite structures and the auxetic structure. (**a**) Young’s modulus, (**b**) yield stress, (**c**) energy absorption capacity and (**d**) specific energy absorption (SEA) are analyzed.

A comparative analysis of the mechanical performance is provided in Figure 7, where Young’s modulus, yield stress, energy absorption, and specific energy absorption (SEA) are evaluated. In all cases, the auxetic-reinforced composites exhibit superior load-bearing capacity and enhanced energy dissipation compared to the corresponding neat resins. This improvement demonstrates a synergistic effect between the polymer matrix and the auxetic geometry under compressive loading, indicating a more efficient stress transfer and deformation mechanism within the composite structure.

In addition, it is possible to observe how the Young’s Modulus and the Yield Strength change in this composite structure. Figure 7 shows the analysis of the mechanical behavior of each structure reinforced with the three different types of resins. On one hand, it is possible to observe that Vinylester Resin exhibits the highest Young’s Modulus, while Epoxy Resin has the lowest Young’s Modulus, with the composite structures following the same trend. Additionally, there is a significant similarity between the Young’s Modulus of the resins and the Young’s Modulus of the composite structures. In the case of the composite structure with Vinylester Resin, the Young’s Modulus decreases by 1.7% compared to the pure resin sample. In contrast, the Young’s Modulus decreases by 10.8% for the composite structure with Polyester Resin and by 26.0% for the composite structure with Epoxy Resin, as shown in Figure 7a. On the other hand, when analyzing the yield stress of the structures, a similar trend is observed. That is, Vinylester Resin shows greater strength, while Epoxy Resin has lower strength.

Although Vinylester provides the highest matrix stiffness among the tested systems, the Vinylester composite exhibited a 37.2% reduction in yield strength relative to the neat resin, whereas Epoxy and Polyester composites increased by 10.0% and 2.0%, respectively (Figure 7b). This behavior is explained by the interaction between resin stiffness, auxetic load-sharing, and stress localization. The Vinylester composite modulus remains nearly equal to that of the neat resin (−1.7%, Figure 7a), indicating that the PLA lattice does not significantly participate in carrying load at small strains. In this case, the introduction of additional PLA–resin interfaces and sharp re-entrant features creates localized stress concentrations and promotes premature microcracking or interfacial debonding, which govern the apparent yield point. Conversely, the lower-modulus, tougher Epoxy and Polyester matrices engage the auxetic lattice earlier in the deformation process, promoting more distributed shear deformation and delaying yield onset. This interpretation is consistent with the significantly greater plastic strain capacities observed for all auxetic-reinforced structures compared to their respective neat resin specimens (Figure 5) and with the known sensitivity of brittle Vinylester systems to geometric and interfacial defects reported in literature. Future investigations will incorporate in situ deformation imaging to directly quantify strain localization and validate the predicted failure mechanisms.

Similarly, a comparable trend can be observed when analyzing the energy absorption capacity. The composite structure with Vinylester resin exhibits a higher capacity, while structures composed of Polyester and Epoxy resins exhibit a lower energy absorption capacity, and their values are very similar to each other. However, in all cases the composite structures manage to surpass the energy absorption of the pure resin samples by approximately 21.7%. This demonstrates the contribution of the auxetic structure in the composite, significantly enhancing its energy absorption capacity, as shown in Figure 7c.

Finally, to compare the mechanical behavior of the auxetic structure with the resin samples and composite structures, the densities of the samples can be incorporated into the analysis (Figure 6). The auxetic structure undergoes significant plastic deformations; however, it achieves low levels of strength due to its porous, low-density nature. The energy absorption capacity of each sample is divided by its density to calculate the specific energy absorption (SEA), as shown in Figure 7d and in accordance with Equation (2). The auxetic structure achieves a SEA value of 1.7 [J/gr]. However, this value is not comparable to the levels reached by composite structures, which can achieve up to 31.2 [J/gr]. Nonetheless, by incorporating the auxetic structure into the resin matrix, the SEA index can improve by up to 26% compared to the pure resin sample.

## 4. Conclusions

This study presented an experimental comparison of auxetic structures reinforced with three different polymeric resin matrices (epoxy, polyester, and vinylester), fabricated by fused deposition modeling (FDM) and some under quasi-static compression. The objective of this investigation arises from the need to improve the energy absorption capacity of FDM-printed auxetic structures, which are limited by weak interlayer bonding. While previous studies have shown that embedding auxetic geometries with polymeric matrices such as foams, mortars, or individual resins improves their mechanical response, no previous research has systematically compared different types of resin matrices within a unified auxetic architecture. This investigation addresses this deficiency and provides new insights into how the type of resin matrix affects the mechanical performance and energy dissipation of the composite. The main findings are as follows:

- All resin-reinforced auxetic composites outperformed both unfilled auxetic structures and pure resin samples in terms of stiffness, strength, and energy absorption, confirming the synergistic interaction between geometry and matrix.
- Compared to pure resin samples, the composite structures showed: a 21.7% increase in energy absorption capacity, up to a 26% increase in specific energy absorption, and a slight decrease in Young’s modulus (1.7% for Vinylester, 10.8% for Polyester, and 26% for Epoxy).
- Decrease in yield strength for Vinylester (37.2%), but increase for Polyester (2%) and Epoxy (10%). These results demonstrate the significant enhancement in mechanical performance achieved by reinforcing auxetic structures with resin matrices, particularly with Vinylester resin, making this novel composite a promising candidate for advanced energy absorption applications.

The study confirmed that resin selection plays a critical role in tailoring the mechanical response of auxetic composites and that the reinforcement process is straightforward to implement, as it involves directly pouring the resin into the open internal geometry of the printed structures. These findings provide a practical route to improving the mechanical behavior of 3D-printed auxetic structures for use in energy-absorbing components, such as crash mitigation systems, protective barriers, or industrial safety devices.

Although this study employed two replicates per configuration due to fabrication constraints, the low variability observed between tests and the clear performance differences among matrices reinforce the reliability of the conclusions drawn.

## Figures and Tables

**Table 1:** Summary of experimental samples manufactured for the compression.

Sample	Compression Test
Auxetic Structure	2
Polyester Resin	2
Auxetic + Polyester Resin	2
Vinylester Resin	2
Auxetic + Vinylester Resin	2
Epoxy Resin	2
Auxetic + Epoxy Resin	2
**Total**	14

## Data Availability

The original contributions presented in this study are included in the article. Further inquiries can be directed to the corresponding authors.
